# A Test-and-Not-Treat Strategy for Onchocerciasis Elimination in *Loa loa*–coendemic Areas: Cost Analysis of a Pilot in the Soa Health District, Cameroon

**DOI:** 10.1093/cid/ciz461

**Published:** 2019-06-04

**Authors:** Edeltraud J Lenk, Henri C Moungui, Michel Boussinesq, Joseph Kamgno, Hugues C Nana-Djeunga, Christopher Fitzpatrick, Anne-Claire M M Peultier, Amy D Klion, Daniel A Fletcher, Thomas B Nutman, Sébastien D Pion, Yannick Niamsi-Emalio, William K Redekop, Johan L Severens, Wilma A Stolk

**Affiliations:** 1 Erasmus School of Health Policy and Management, Erasmus University Rotterdam; 2 Department of Public Health, Erasmus Medical Center, University Medical Center Rotterdam, The Netherlands; 3 Centre for Research on Filariasis and Other Tropical Diseases, Yaounde, Cameroon; 4 Unité Mixte Internationale, TransVIHMI, Institut de Recherche pour le Développement, University of Montpellier, France; 5 Neglected Tropical Diseases, World Health Organization, Geneva, Switzerland; 6 National Institute of Allergy and Infectious Diseases, National Institutes of Health, Bethesda, Maryland; 7 Department of Bioengineering, University of California, Berkeley

**Keywords:** onchocerciasis, *Loa loa*, cost analysis, point-of-care testing, disease elimination

## Abstract

**Background:**

Severe adverse events after treatment with ivermectin in individuals with high levels of *Loa loa* microfilariae in the blood preclude onchocerciasis elimination through community-directed treatment with ivermectin (CDTI) in Central Africa. We measured the cost of a community-based pilot using a test-and-not-treat (TaNT) strategy in the Soa health district in Cameroon.

**Methods:**

Based on actual expenditures, we empirically estimated the economic cost of the Soa TaNT campaign, including financial costs and opportunity costs that will likely be borne by control programs and stakeholders in the future. In addition to the empirical analyses, we estimated base-case, less intensive, and more intensive resource use scenarios to explore how costs might differ if TaNT were implemented programmatically.

**Results:**

The total costs of US$283 938 divided by total population, people tested, and people treated with 42% coverage were US$4.0, US$9.2, and US$9.5, respectively. In programmatic implementation, these costs (base-case estimates with less and more intensive scenarios) could be US$2.2 ($1.9–$3.6), US$5.2 ($4.5–$8.3), and US$5.4 ($4.6–$8.6), respectively.

**Conclusions:**

TaNT clearly provides a safe strategy for large-scale ivermectin treatment and overcomes a major obstacle to the elimination of onchocerciasis in areas coendemic for *Loa loa*. Although it is more expensive than standard CDTI, costs vary depending on the setting, the implementation choices made by the institutions involved, and the community participation rate. Research on the required duration of TaNT is needed to improve the affordability assessment, and more experience is needed to understand how to implement TaNT optimally.

## Teaser

We measured the costs of a community-based pilot using a safe test-and-not-treat strategy to treat onchocerciasis with ivermectin in areas coendemic for *Loa loa.* Costs per person were US$4.0 (population), US$9.2 (tested), and US$9.5 (treated).

Community-directed treatment with ivermectin (CDTI) has been used since 1999 as the main strategy to combat onchocerciasis in Africa [1, 2]. While generally considered safe, ivermectin has been associated with severe adverse events (SAEs) in individuals with high levels of circulating microfilariae (mf) of *Loa loa* (another filarial parasite endemic in Central Africa) [3, 4]. CDTI has been implemented in coendemic areas where the proportion of individuals with *Onchocerca volvulus* nodules exceeds 20% (hyperendemic and mesoendemic areas), albeit with enhanced adverse event (AE) surveillance. However, fear of SAEs has caused individuals in such areas to refuse treatment, leading to suboptimal drug coverage and continued *O. volvulus* transmission. Moreover, CDTI is not recommended in *Loa*-coendemic areas where *O. volvulus* is hypoendemic (nodule prevalence <20%), as the risk of SAEs is thought to outweigh the benefits of CDTI. This jeopardizes onchocerciasis elimination in Africa [5, 6].

A test-and-not-treat (TaNT) strategy using a smartphone-based videomicroscope (LoaScope) enabled the safe implementation of ivermectin treatment in a pilot study in the Okola health district (HD) in Cameroon where all individuals ≥5 years of age [7, 8] were included. Of 16 259 tested individuals, only 340 (2.1%) were excluded from ivermectin treatment because of microfilarial counts >20 000 mf/mL, whereas 15 522 (95.5%) were treated without the occurrence of SAEs [7]. A second TaNT study in Soa HD also had no postivermectin SAEs and further demonstrated that TaNT could be performed by local health workers and community members under the supervision of the research team [9]. Additional information about this TaNT round in the Soa HD can be found in Supplementary Appendix 1.

Although TaNT is a promising strategy for onchocerciasis elimination in loiasis-coendemic areas, its implementation is expected to be costlier than that of CDTI. Consequently, there are affordability concerns at a wider scale. Thus, we measured the cost of community-based TaNT in the above-mentioned Soa pilot study [9]. In addition, we estimated the cost for 3 programmatic implementation scenarios (base-case, less intensive, and more intensive), to obtain a range of plausible estimates under different assumptions of resource use.

## METHODS

### General Approach

A microcosting approach [10] was used to empirically assess the cost of a community-based TaNT in the Soa HD [9]. The empirical costing study reflects the costs borne by the organizations involved in implementing and executing the program in this specific pilot. A future program would likely be run by the Cameroonian Ministry of Health (MoH) in partnership with nongovernmental development organizations (NGDOs). We aimed to reflect this future situation in our cost calculations and therefore calculated the economic costs of the pilot, including financial costs (explicit cash expenditures on project activities) and added relevant opportunity costs (resources that could be used in other projects, if not used in this one) [11]. We designed the protocol, data collection instruments, and cost calculation sheets following standard guidelines and reported input quantities and costs to maximize comparability with other studies and recently published mass drug administration (MDA) benchmarks [11, 12].

### Study Area and Population

**Figure 1.**
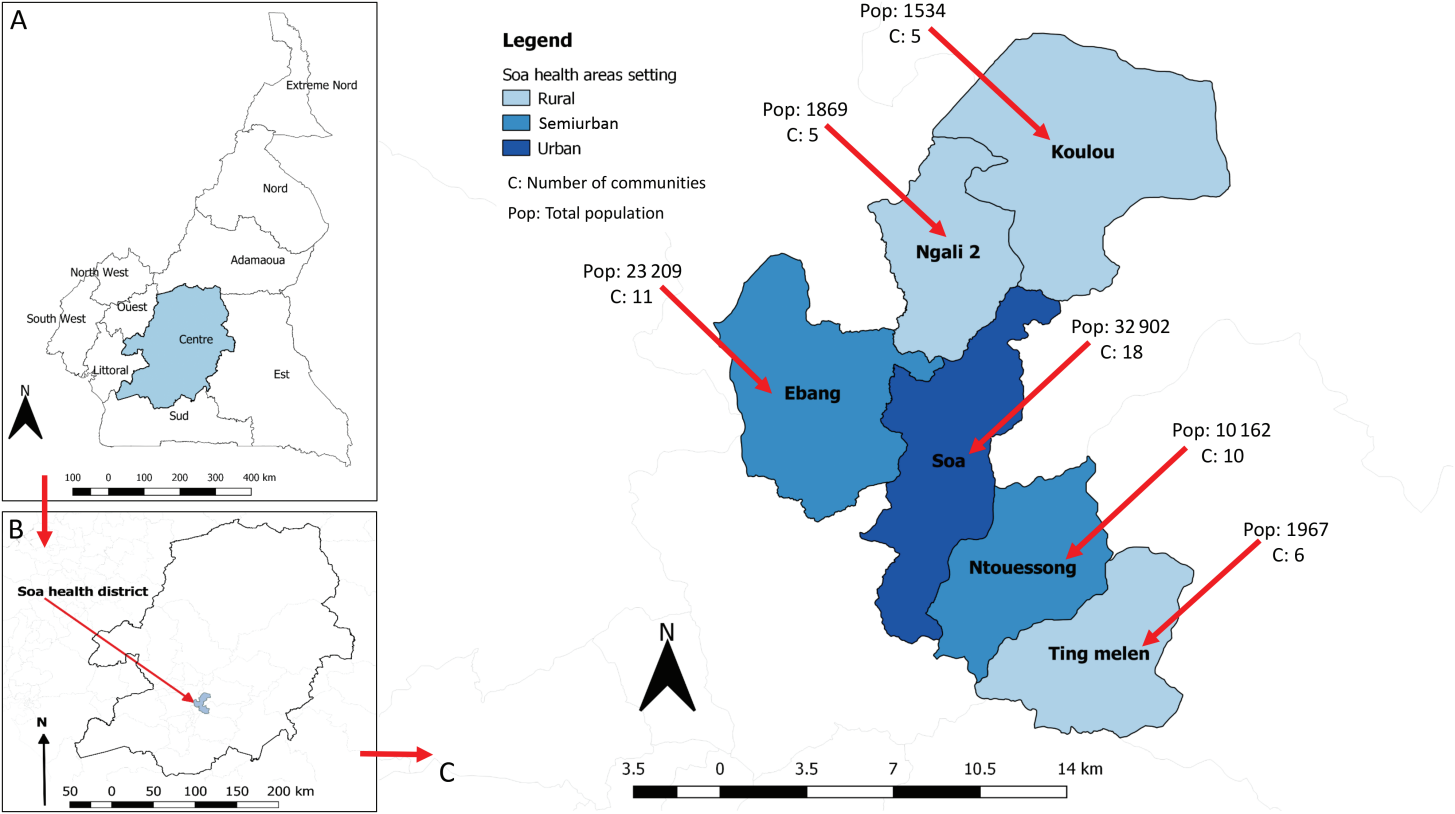
Map of Soa health district in Cameroon, showing the rural/urban characterization, number of communities, and population size by health area.

The Soa HD in Cameroon (Figure 1) is an ivermectin-naive district that is hypoendemic for onchocerciasis and coendemic for loiasis [13]. It is located 17 km from Yaoundé (Cameroon’s capital) and is easily accessible. Soa HD consists of 6 health areas (3 rural, 2 semiurban, 1 urban) and its population was estimated at 71 643 inhabitants, according to the census performed as part of the TaNT pilot.

### Time Window

The census, testing/treating, and monitoring of SAEs in the TaNT round took place from September 2017 to February 2018. Costs were collected beginning with the preparation of the round in August 2017 until the last payments in March 2018.

### Cost Calculation Components

The “supplies” category includes (but is not restricted to) fuel, car maintenance, food, transportation, office materials, field materials, and drugs to treat AEs. The “personnel” category includes per diems of community drug distributors (CDDs) and other staff, project staff salaries, and fringe costs for study personnel, when applicable. Overhead costs were not explicitly measured but were assumed to be 15% of direct costs, as reported in the project budget. Supplementary Appendices 1 and 2: Tables 1, 2, and 4) provide additional information. We excluded the following costs: ivermectin costs (as the drug was donated); bank tariffs; management/administrative costs incurred outside the country; costs incurred by households to be treated; opportunity cost of time spent by school staff; and opportunity costs of buildings used by MoH staff and of capital items purchased for previous projects.

Costs of LoaScopes and capillaries were provided in US dollars (US$); all other costs were reported in Central African francs (XAF) and converted to US$ using the US Treasury Reporting Rates of Exchange of 31 December 2017 (US$1 = XAF 568) [14].

### Data Collection

Costs were based on actual payments or booked transfers collected from all invoices and bank transactions provided by project managers. External MoH staff working in the project and CDDs were interviewed using structured questionnaires to obtain information about their time spent on the project and opportunity cost of their salaries. A summary of the design, structure, and dissemination protocol for each questionnaire and English versions of these can be found in Supplementary Appendix 3: Table 6 and Questionnaires 1–3).

### Data Entry, Cleaning, and Analysis

The invoices were analyzed and compared to the data provided by monthly financial reports and bank transactions for consistency and quality checks. The value of each invoice or bank transaction was labeled according to the input category, the health area in which the cost was incurred (using the label “district costs” if costs were not specific to any health area, but relative to the project as a whole), and the activity to which the cost could be attributed (advocacy, census, planning and budgeting, procurement, training, health education in the community and mobilization [HECM], delivery and distribution of interventions to target populations, AE surveillance and management, monitoring and evaluation, and general management). Data from invoices and questionnaires were entered into a Microsoft Excel spreadsheet for further analysis. Overhead costs were added to the subtotal and the final result was divided by the project outputs.

### Scenario Analysis

To assess the effect of different implementation strategies on the overall cost of TaNT, costs were calculated using 3 hypothetical alternative TaNT implementation scenarios: “base-case,” “less intensive resource use,” and “more intensive resource use.” The scenarios were designed based on the literature and our own expertise in conducting CDTI in Cameroon, using the same HD for context (eg, applying the same rural/urban distribution, same distances to the capital, same mode of delivery) [2, 15]. Table 1 describes the main differences between the scenarios and the pilot. See Supplementary Appendix 4: Tables 7–9) for a more detailed description.

**Table 1.** Summarized Description of Alternative Scenarios

	Cost Scenario		
Cost Category	Base-case	Less Intensive Resource Use	More Intensive Resource Use
Personnel			
Supervision and M&E	<< pilot Smaller teams and fewer days than pilot	<<< pilot Same team and fewer days than base-case	< pilot Bigger team and more days than base-case
HECM	< pilot (no sound car^a^)	< pilot (no sound car^a^)	< pilot (no sound car^a^)
AE surveillance and management	Same as pilot	<< pilot (50% less than pilot)	Same as pilot
CDDs	< pilot (CDDs only paid for training days)	< pilot (CDDs only paid for training days)	>> pilot (CDDs were paid per diems for treatment days corresponding to the average income of an 8-h workday (to account for income loss)
Blood drawers and loascopists	> pilot (were paid a higher transport fee per field day, to account for more distant communities: based on responses of questionnaires)	> pilot (same as base-case)	>> pilot (higher transport fees than base-case)
School workers	None	None	100 workers
Supplies			
Fuel	> pilot (extra fuel allowance for MoH and NGDO cars)	> pilot (same as base-case)	>> pilot (higher allowances than base-case)
LoaScopes and capillaries	< pilot (assumed large-scale prices)	< pilot (same as base-case)	Same as pilot
Other consumables	< pilot (30% less than pilot)	< pilot (50% less than pilot)	Same as pilot

Abbreviations: <, less than; <<, lesser than; <<<, much lesser than; >, more than; >>, much more than; AE, adverse events; CDD, community drug distributors; HECM, health education in the community and mobilization; M&E, monitoring and evaluation; MoH, Ministry of Health; NGDO, nongovernmental development organization.

^a^Megaphone-equipped car.

## RESULTS

The population size and numbers of individuals tested, treated, or excluded, as well as the occurrence of AEs, per health area are given in Table 2. Only 0.8% of the tested population was excluded from treatment. The overall coverage was relatively low (42%).

**Table 2.** Program Outputs (Number of Individuals)

Health Area	Censused	Censused Aged >5	Tested With LoaScope	Treated	Excluded for *Loa* >20 000 mf/mL	Excluded for Other Reasons^a^	AEs^b^	Coverage (Treated/ Censused), %
Ting Melen (r)	1967	1697	1644	1585	16	43	7	81
Koulou (r)	1534	1342	1035	975	38	22	18	64
Ngali 2 (r)	1869	1594	1017	963	36	18	19	52
Ebang (su)	23 209	19 906	11 412	10 987	53	372	47	47
Ntouessong (su)	10 162	8618	3602	3473	44	85	25	34
Soa (u)	32 902	29 524	12 098	11 765	58	275	68	36
Total	71 643	62 681	30 808	29 748	245	815	184	42

Abbreviations: AE, adverse event; mf, microfilariae; r, rural; su, semiurban; u, urban.

^a^Pregnant and breastfeeding women, individuals suffering from chronic disease.

^b^Number of AEs (multiple AEs may occur in 1 person).

The costs of the pilot, disaggregated by programmatic activity, are presented in Table 3. The costliest activities were related to the actual delivery of the intervention (31%) and HECM (11%), although general management costs (not related to a specific activity) also formed a large part of the total costs (19%). The supplies input categories that were responsible for the highest shares of the total costs (from highest to lowest), are capillaries and information, education, and communication materials leading with 12% and 10% of the total pilot costs, respectively (Supplementary Appendix 2: Table 3). Supplementary Appendix 5: Table 10 shows volumes and prices of supplies per input category.

**Table 3.** Total Costs of Pilot per Activity (US Dollars)

Program Activity	Supplies	Personnel	Total	Percentage of Total Pilot Costs
1. Advocacy	6020	843	6864	2%
2. Census	741	18 975	19 715	7%
3. Planning and budgeting	100	8071	8170	3%
4. Procurement	30	9	39	0.01%
5. Training	2537	13 542	16 078	6%
6. HECM	21 442	8979	30 421	11%
7. Delivery intervention	46 527	42 137	90 115	31%
8. AE surveillance and management	2048	10 253	12 300	4%
9. M&E	107	11 606	11 712	4%
10. General management^a^	18 280	34 613	52 892	19%
Subtotal activities	97 830	149 027	248 308	87%
Overhead costs	^b^	^b^	37 253^b^	13%
Total	112 504	171 381	283 885	100%

Abbreviations: AE, adverse event; HECM, health education in the community and mobilization; M&E, monitoring and evaluation.

^a^Includes all inputs related to the project as a whole and that could not be attributed to a specific activity (eg, electricity, some office supplies, communication, some of the fuel and car maintenance costs).

^b^Calculated as 15% of the activities subtotal.

The majority of the personnel costs were not related to a specific activity but to actions related to the implementation of the round as a whole. The same holds true for district and health area level MoH personnel. The main costs of all other categories were related to the delivery of the intervention (Supplementary Appendix 2: Table 5).

The total cost for the entire pilot was US$283 938 (Table 4), with a cost per person treated of US$9.5. The cost per person treated was reduced to US$5.4 for the base-case and to US$4.6 and US$8.6 for the less and more intensive scenarios, respectively.

**Table 4.** Total Costs per Round and per Program Output (US Dollars): Pilot and Alternative Scenarios

Health Area and Scenario	Total Costs of the Pilot per Health Area	Total No. Censused	Cost per Person Censused	Cost per Person Tested	Cost per Person Treated	Coverage (Treated/Censused), %
Empirical cost estimates by health area						
Ting Melen (r)	17 770	1967	9.0	10.8	11.2^a^	81^b^
Koulou (r)	14 994	1534	9.8	14.5	15.4^a^	64^b^
Ngali 2 (r)	16 161	1869	8.6	15.9	16.8^a^	52^b^
Ebang (su)	90 425	23 209	3.9	7.9	8.2	47^c^
Ntouessong (su)	41 163	10 162	4.1	11.4	11.9	34^c^
Soa (u)	103 425	32 902	3.1	8.5	8.8	36^c^
Total costs of the TaNT pilot	283 938	71 643	4.0	9.2	9.5	42
Alternative implementation scenarios						
Base-case	159 349	71 643	2.2	5.2	5.4	42
Less intensive	137 289	71 643	1.9	4.5	4.6	42
More intensive	255 850	71 643	3.6	8.3	8.6	42

Abbreviations: r, rural; su, semiurban; TaNT, test and not treat; u, urban.

^a^Rural areas had a higher cost per person treated due to more supervisory personnel (they were the first areas to participate in the pilot), more fuel costs (increased distance from the capital), and additional testing/treating at schools.

^b^Rural areas had higher coverage because people are at home or work close to home and can be reached more easily by community drug distributors during the sensitization phase and because people are available during sampling hours (sampling needs to be done during regular school/work hours [between 10:00 am and 4:00 pm] due to the diurnal periodicity of *Loa loa* microfilaremia).

^c^Semiurban and urban areas had lower coverage because of more treatment refusals and the absence of people at work/school during sampling hours.

**Figure 2.**
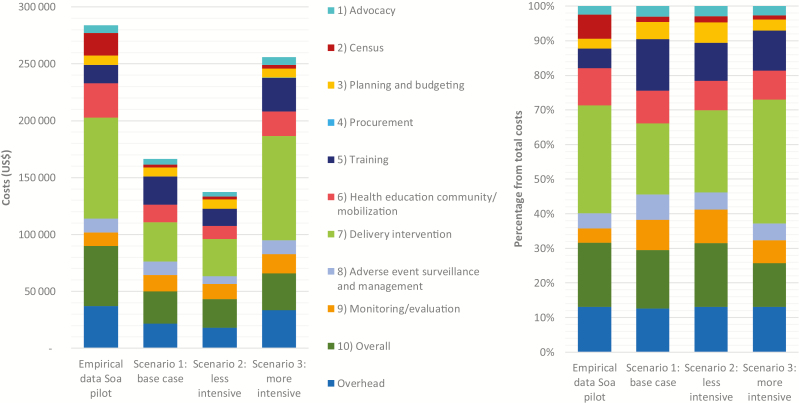
Total costs: empirical vs scenarios.

The absolute costs of the Soa pilot and each of the implementation scenarios disaggregated by programmatic activity, as well as the percentages of each activity, are given in Figure 2. The empirical study was more expensive than the scenarios, mainly due to the census, HECM, general management costs (for instance car maintenance and field materials), and the costs of LoaScopes and capillaries. Costs of delivering the intervention formed the largest share of the total costs, mainly due to the costs of capillaries, field material, and per diems, which showed a reduction in the base-case and less intensive scenarios mostly due to less supervision and less expensive materials. Costs increased considerably in the more intensive scenario, due to the payment of higher per diems to CDDs to account for income loss. Even though general management costs were also reduced by less expensive materials in the scenarios, the percentage of the total costs did not change much. Costs of HECM were lower in the scenarios because of the exclusion of the costs of the megaphone-equipped car and the reduction of prices of materials.

## DISCUSSION

This study provides both empirical cost estimates of a pilot and projected costs based on scenario analyses using the new TaNT strategy in regions coendemic for *O. volvulus* and *L. loa.* Based on actual expenditure during the pilot, this strategy cost US$4.0 per person in the population, US$9.2 for each person tested, and US$9.5 for each person treated. In the alternative implementation scenarios, these costs (shown as base-case estimates with less and more intensive scenarios) were estimated to be US$2.2 ($1.9–$3.6), US$5.2 ($4.5–$8.3), and US$5.4 ($4.6–$8.6), assuming that population participation remained unchanged.

Several limitations apply to the internal validity of our study. Despite the careful collection of available data from invoices, bank transfers, and questionnaires, we had to adapt to the system usually used by financial managers of the implementing institution, without explicit reporting of the cost of previously acquired capital items or building rental. Using a retrospective questionnaire investigating opportunity costs of MoH staff was also not ideal, as it allows recall bias. Opportunity costs of time spent by school staff when helping during the treatment phase and of vehicles and furniture acquired for other projects were not included in this study, leading to a slight underestimation of the costs of the pilot. These limitations should be addressed in future studies to increase the accuracy of cost estimates and enable policymakers to anticipate their costs more reliably. This would also allow economic costs to be presented separated from financial costs.

Because we measured costs in a study setting, alongside a pilot implementation of community-based TaNT, our results may not be fully representative of a programmatic setting, limiting the external validity. Some costs may have been higher than expected under programmatic implementation (eg, all personnel received intensive training and supervision and CDDs received payment). Furthermore, best implementation practice still remains to be determined, and future programs may be implemented somewhat differently. The programmatic operationalization scenarios were designed to address the external validity limitations. They still do not cover all possible variations in implementation choices. We built the base-case scenario aiming to reflect Cameroonian reality as much as possible. As shown in the scenario analyses, costs heavily depend on implementation choices, and cost-savings compared to the pilot can be achieved through reductions in supervisory personnel, health education and sensitization, surveillance and management of AEs, and purchase prices of the LoaScopes and capillaries. The last 2 categories can be further reduced by implementing NGDOs/MoH if external funding agencies opt to donate these supplies to countries choosing to implement the TaNT strategy, as already done with the donation of ivermectin.

Overall study coverage was relatively low, particularly in the semiurban and urban areas. Higher coverage is essential to onchocerciasis elimination. Achieving high coverage in urban areas is a known problem, even in standard CDTI, due to lack of trust in public programs, migrant populations, and disorganized poor urban settlements [16, 17]. Yet, it may be even more difficult to achieve in TaNT programs, as blood sampling needs to be done during regular school/work hours, requires the involvement of additional people (blood drawers and “loascopists”), and is best done with a mobile station rather than house-to-house. Additional measures are needed to increase coverage, including continuous and improved health education/mobilization and alternative ways to offer testing and treatment, such as mobile stations at schools and in commercial/industrial areas. However, because it is difficult to predict the cost and impact of these measures on coverage, we have not considered increases in coverage in our implementation scenarios. Although there will be some economies of scale (fixed costs divided over a larger population), other costs are likely to increase with the number of people covered (eg, personnel costs for delivering the intervention), resulting in a higher cost per person.

Our estimates, derived from a community-based single study, are not directly transferable to other settings due to many factors, such as variations in local prices, organization and availability of healthcare, and geographic characteristics such as remoteness, accessibility, and level of “urbanness” [18, 19].

To eliminate onchocerciasis, treatment would have to be repeated yearly (or more frequently) for many years with sufficient treatment coverage (ie, 80% of the eligible population per World Health Organization guidance) [20]. The costs of future rounds are likely to be significantly lower than the initial round for a number of reasons, including the absence of startup costs, shared fixed costs (over multiple rounds within a given year), more efficient implementation (from the experience and structure provided by the first round), lower intensity of HECM (communities would already be aware of the strategy), and less MoH advocacy and AE surveillance (since fewer cases would need to be followed). Further, as *L. loa* microfilarial densities remain low after treatment for at least 18 months, people proven to be treated in the previous round (using reidentification methods) would not need to be retested [21]. Capillary and LoaScope costs would only remain for the individuals not previously tested and treated, making costs much more comparable to those of standard CDTI.

To date, there are no studies of the costs of alternative strategies of measuring *L. loa* microfilarial densities before treating for onchocerciasis to which we could compare our results. We cannot directly estimate the difference in costs between TaNT and CDTI without empirical comparisons, as it is impossible to disentangle costs of treatment from those of testing in the TaNT strategy. A unit cost benchmarking study by Fitzpatrick et al [11] showed that the unit cost of mass drug administration is highly variable, depending very much on the use of volunteers and economies of scale. This variation complicates a direct comparison of our results with any previously published figures. A web-based tool [11] that provides unit cost benchmarks for different settings allowed us to estimate the expected cost of a first round of standard CDTI in Soa, using the corresponding population and characteristics of the area (which the tool allows), and the resulting cost estimate was US$18.3 (95% confidence interval [CI], $6.9–$35.6) per person treated using paid community health workers. The observed cost of TaNT for the pilot and more intensive scenario are closer to the lower end of the range despite the cost of testing. The benchmark unit cost of standard CDTI is considerably lower when implemented by unpaid community volunteers, US$3.9 (95% CI, $2.2–$5.7), and the cost of our base-case and low-intensity implementation scenario comes quite close to this. Details on the use of the benchmark tool are provided in Supplementary Appendix 6.

Through TaNT, community treatment with ivermectin can be expanded into the many areas in central Africa that are hypoendemic for onchocerciasis and coendemic for loiasis, where standard CDTI cannot be applied for safety reasons. Implementing treatment in these regions is crucial for the continent-wide elimination of onchocerciasis, with associated health and socioeconomic benefits estimated at more than US$6 billion [22]. The populations concerned will not only benefit from the control and potential elimination of onchocerciasis but—as ivermectin is a broad-spectrum anthelmintic drug—also from the effects of treatment of other parasitic diseases including scabies and soil-transmitted helminthiases.

## CONCLUSIONS

Costs of TaNT are higher than costs of standard CDTI. How much higher will depend on how subsequent treatment rounds will be implemented and the required program duration. The duration will not be much longer than in *Loa*-free areas if the coverage is sufficiently high and the proportion of the population excluded from treatment (because of high-density loiasis) is as low as observed in Soa and Okola (1% on average). The effect of excluding people from treatment may be more important where the proportion of people excluded due to loiasis is higher, but this can be mitigated by offering alternative treatment for onchocerciasis, such as a course of doxycycline. Although we did not assess the costs associated with such treatment, these should be weighed against the impact on program duration and success.

Even though TaNT is undoubtedly more expensive than MDA, onchocerciasis elimination will not be reached without a strategy that can be safely used in areas coendemic for *L. loa*. Our empirical study shows that TaNT using LoaScopes remains affordable, given the enormous potential economic benefits of reaching elimination of onchocerciasis in Cameroon and in other countries where *L. loa* is coendemic with *O. volvulus.* Various implementation scenarios show that costs could be further reduced. Future studies in this field are needed to investigate the costs of a programmatic implementation.

## Supplementary Data

Supplementary materials are available at *Clinical Infectious Diseases* online. Consisting of data provided by the authors to benefit the reader, the posted materials are not copyedited and are the sole responsibility of the authors, so questions or comments should be addressed to the corresponding author.

ciz461_suppl_Supplementary_Material

## Notes

***Acknowledgments.*** The authors express their true appreciation to Ann Goldman, Paul Cantey, Sim Azebaze, Raoul Ghomsi, and Katia Urena, whose important comments and insights contributed to this work.

***Financial support.*** This work was supported by the Bill & Melinda Gates Foundation (grant number OPP1033740 to M. B., J. K., H. C. N., A. D. K., T. B. N., D. A. F., S. D. P., Y. N., and W. A. S.) and the Division of Intramural Research, National Institute of Allergy and Infectious Diseases, National Institutes of Health (to A. D. K. and T. B. N.). E. J. L. is external PhD at the Erasmus School of Health Policy and Management and this affiliation is formalized by means of an unpaid hospitality agreement. E. J. L. has performed the work for this study as external junior researcher, formalized by a paid fixed-term collaboration agreement with the Department of Public Health, Erasmus Medical Center, Rotterdam.

***Potential conflicts of interest.*** D. A. F. is an inventor on patents related to mobile phone imaging: High Numerical Aperture Telemicroscopy Apparatus (US patents 8743194 and 8786695) licensed by Thermo Fisher Scientific and CellScope Inc. All other authors report no potential conflicts of interest. All authors have submitted the ICMJE Form for Disclosure of Potential Conflicts of Interest. Conflicts that the editors consider relevant to the content of the manuscript have been disclosed.
