# Supplementary material for: A Test-and-Not-Treat Strategy for Onchocerciasis Elimination in Loa loa–coendemic Areas: Cost Analysis of a Pilot in the Soa Health District, Cameroon
Source: Clin Infect Dis. 2019 Jun 4;70(8):1628–35. doi: 10.1093/cid/ciz461 (PMC7146010; doi:10.1093/cid/ciz461)
Supplement: ciz461_suppl_Supplementary_Material [file ciz461_suppl_supplementary_material.docx]

**Supplementary data**

# **A Test-and-Not-Treat strategy for onchocerciasis elimination in *Loa loa* co-endemic areas: cost analysis of a pilot in Soa health district, Cameroon**

Edeltraud J Lenk*, Henri C Moungui, Michel Boussinesq, Joseph Kamgno, Hugues C Nana-Djeunga, Christopher Fitzpatrick, Anne-Claire MM Peultier, Amy D Klion, Thomas B Nutman, Daniel A Fletcher, Sébastien D Pion, Yannick Niamsi-Emalio, William K Redekop, Johan L Severens, Wilma A Stolk.

* Corresponding author: Edeltraud Johanna Lenk, Erasmus School of Health Policy and Management, Erasmus University Rotterdam, P.O. Box 1738, 3000 DR Rotterdam, The Netherlands. E-mail: [lenk@eshpm.eur.nl](mailto:lenk@eshpm.eur.nl)

Contents

[A Test-and-Not-Treat strategy for onchocerciasis elimination in *Loa loa* co-endemic areas: cost analysis of a pilot in Soa health district, Cameroon 1](#_Toc8725613)

[Appendix 1. Summarized description of the TaNT pilot in Soa HD 3](#_Toc8725614)

[Table S1. Activities in standard CDTI in Cameroon, pilot of community-based TaNT in Soa HD, and TaNT base case implementation scenario 4](#_Toc8725615)

[Appendix 2. Input categories 7](#_Toc8725616)

[Table S2. Inputs of supplies categories 7](#_Toc8725617)

[Table S3. Costs of supplies responsible for the highest shares of the total costs disaggregated by input category and activity (US$) 8](#_Toc8725618)

[Table S4. Inputs of personnel categories 9](#_Toc8725619)

[Table S5. Costs of personnel disaggregated by category and activity (US$) 10](#_Toc8725620)

[Appendix 3. Questionnaires 12](#_Toc8725621)

[Table S6. Design, structure and dissemination of the questionnaires for CDDs and external staff from the Ministry of Health. 12](#_Toc8725622)

[Appendix 4. Scenarios 16](#_Toc8725623)

[Table S7. Assumptions regarding differences in personnel costs between the base case, less intensive and more intensive resource use implementation scenarios. Costs are specified in *Communauté Financière Africaine* (CFA, African Financial Community) francs (XAF). 16](#_Toc8725624)

[Table S8. Assumptions regarding differences in personnel per diems between the base case, less intensive and more intensive resource use implementation scenarios. Costs are specified in *Communauté Financière Africaine* (CFA, African Financial Community) francs (XAF). 19](#_Toc8725625)

[Table S9. Assumptions regarding differences in supplies costs between the base case, less intensive and more intensive resource use implementation scenarios. Costs are specified in *Communauté Financière Africaine* (CFA, African Financial Community) francs (XAF). 21](#_Toc8725626)

[Appendix 5. Volumes of supplies input categories 23](#_Toc8725627)

[Table S10. Volumes of supplies per input category. Prices specified in *Communauté Financière Africaine* (CFA, African Financial Community) francs (XAF). 23](#_Toc8725628)

[Appendix 6. Benchmark tool use 25](#_Toc8725629)

[References 25](#_Toc8725630)

##

## **Appendix 1. Summarized description of the TaNT pilot in Soa HD**

Cameroon participated in the African Programme for Onchocerciasis Control (APOC) between 1995 and 2015, adopting CDTI as its main strategy to control onchocerciasis morbidity in meso- and hyperendemic areas.[1,2] During this period, Cameroon reported 471 SAEs following ivermectin treatment (~34% of all reported cases in the Mectizan Donation Program). Encephalopathy was reported in 221 cases, mostly (90%) in individuals taking ivermectin for the first time.[3]

This pilot was implemented in the Soa Health District (HD) by the Centre de Recherche sur les Filarioses et autres Maladies Tropicales (CRFilMT), Cameroon in partnership with Institut Bouisson-Bertrand, France and was funded by the Bill and Melinda Gates Foundation (BMGF).

The round started with advocacy meetings, followed by health education and community mobilization about the importance and the dates of the upcoming TaNT-related activities. Community-directed distributors (CDDs) were selected within their own villages (approximately 1/100 inhabitants), whereas blood drawers and loascopists (those responsible for handling the LoaScope) were selected at the level of the health area. All three categories were trained by professionals from the implementing institution and the Ministry of Public Health (MoH). After the training, CDDs conducted village censuses and reported the population numbers to the implementing institution.

After completion of the training, the community teams spent several days in each village to test and treat the population. Inhabitants were invited to come to a central location in the village between 10am and 4pm (because of the diurnal periodicity of *Loa loa* mf in peripheral blood), where the team assessed whether they met the participation criteria (age ≥ 5 years, non-pregnant and without severe chronic diseases) and tested those eligible for participation. The entire eligible population was offered blood screening using a smartphone-based microscope called LoaScope, which provides an accurate estimate of *L. loa* mf counts within 2 minutes.[4] Tested individuals with a count lower than 20,000 *Loa* mf/mL were treated with ivermectin. Those with ≥20,000 *Loa* mf/mL - considered at risk of SAEs - received albendazole for deworming (a widely used, broad-spectrum anthelmintic drug that has no direct effect on *L. loa* mf). The threshold used was lower than the 30,000 mf/mL suggested by the WHO to ensure the prevention of marked adverse events that could raise concern and reduce participation rates.[5,6] Additional testing and treating was performed in schools as a means to increase the coverage among children who were unable to attend the centralized treatment conducted in their communities. A team from the implementing institution (1 physician, 1 pharmacist, 1 nurse, 1 driver) supervised AE surveillance. The surveillance period comprised the first three consecutive days, and day 7 after treatment of each individual. A more detailed description of all activities undertaken for the pilot of community-based TaNT in Soa HD is provided in Table S1, with comparison to standard CDTI and the implementation of a TaNT base case scenario. The scenarios are described in more detail in Appendix 4.

Table S1. Comparison between the activities usually carried out in a standard CDTI round in Cameroon, the pilot of community-based TaNT in Soa HD, and the TaNT base case implementation scenario.[7,8]

###

### **Supplementary Table S1. Activities in standard CDTI in Cameroon, pilot of community-based TaNT in Soa HD, and TaNT base case implementation scenario**

| **Activities** | **Standard local CDTI** | **Community-based TaNT pilot in Soa health district** | **Operational TaNT base case scenario** |
| --- | --- | --- | --- |
| 1. Advocacy | Advocacy meetings (one day each) with key stakeholders and opinion leaders at national, regional and district levels, also at communities, aiming to raise awareness on the campaign and solicit their engagement to support the intervention. | Same as in CDTI. In addition, big launching ceremony presided by the Minister of Health with the participation of various political, administrative and traditional authorities, to introduce a new strategy to the population in general. | Same as in CDTI. |
| 1. Census | Enumeration of the population and acquisition of demographic information by CDDs through a house-to-house approach at their own pace within 1 or 2 weeks, with no per diems paid. | Same as in CDTI, but completed within 3 days, with a financial incentive paid to CDDs. | Same as in CDTI. |
| 1. Planning & budgeting | Planning and budgeting for all activities per CDTI round, through preparatory meetings at central and regional levels and through visits to chiefs of villages (for drawing a list of CDDs). | Same as in CDTI. | Same as in CDTI. |
| 1. Procurement | Order and purchase of drugs, equipment and supplies. Joint drug application package meeting held at central level to define necessary amount of ivermectin (Mectizan®), provided via WHO. | Same as in CDTI, with additional procurement of materials for TaNT. LoaScopes and capillaries. Other materials for field work (cotton, alcohol, tablecloth, bin bags, etc.) purchased locally by the implementing institution. | Same as in TaNT pilot in Soa, with cheaper LoaScopes and capillaries (due to large-scale production) and cheaper field material (purchased at reduced prices by the MoH). |
| 1. Training | Training for any aspect of CDTI for health system employees and community volunteers through cascade training at regional (1 day), district (1 day), health area levels (2 days), CDDs (2 days). | Same as in CDTI, but also including training for blood drawers and loascopists (2 days) and spending 1 day for national level and 2 days for district level training. | Same as in TaNT pilot in Soa. |
| 1. Health education/ community mobilization | Formal and informal education, information and communications about the intervention through different methods. Mass media campaign: radio/TV broadcasting of sensitization messages about the campaign. CDDs deliver health education messages during census and treatment to population, using flyers, posters, t-shirts, banners, and/or calendars.  If specific issues hinder mass drug administration (MDA) coverage in an area, community meetings might be organized, facilitated by health area personnel and supervised by district and regional level staff and staff from NGDO involved in the program. | Same as in CDTI, with the additional use of a sound car (4x4 pick up car specially tuned and equipped with megaphone) manned by one sensitization team composed of one driver, one former NOCP coordinator (team lead) and one team member from CRFilMT. In each health area, this team worked at least 2 days before the census started and then all the period till the treatment ended in the health area. | Same as in CDTI. |
| 1. Delivery of intervention (Treatment) | House-to-house case identification and treatment by CDDs at their pace within 1 or 2 weeks, with no per diems paid. | Testing and subsequent treatment offered at mobile stations in villages by teams consisting of a CDD, a LoaScopist and a blood drawer, within 3 days. CDDs are paid per diems. | Same as in TaNT pilot in Soa, but with per diems paid to CDDs only for training days. |
| 1. Adverse event surveillance and management | Field and clinical monitoring and management of Adverse Events (AEs) and Severe Adverse Events (SAEs); field supervision of this activity. AEs are managed by health facilities at health area or district level, with patients paying for hospitalization, drugs and consultation fees. In case of a SAE, a technical team might go to see the patient and will cover the fees for healthcare. | A team from the implementing institution (1 physician, 1 pharmacist, 1 nurse, 1 driver) was in charge of AE surveillance. They started field surveillance in each health area 3 days after treatment had started and continued until 3 days after the treatment had ended in that health area. They took turns when going to the field. Drugs for AEs management (paracetamol, chlorpheniramine) and medical equipment (tensiometer, stethoscope, etc.) were purchased by implementing institution. | Same as in TaNT pilot in Soa, but with drugs and equipment purchased at reduced prices by the MoH. |
| 1. Monitoring & evaluation (M&E) | Coverage surveys or epidemiological surveys, data quality assessment surveys, specific training on M&E tools (database) not necessarily involving CDDs.  Data collection, data analysis and reporting: after census/treatment, CDDs bring back registers with data. Chief of each health area works about 5 days synthesizing data from the registers; district team (2 persons) then compiles it in Excel for 3-5 days; same happens at regional level. Besides, usually each level spends 3-5 days supervising data collection in the field (including national level and participating NGDO). The NGDO also helps with a data manager. | The implementing institution took care of the entire data entry and management of Soa data. | Same as in CDTI, but with treatment data provided by both written registers and electronic tools. |
| 1. General   Management |  | Inputs that were related to the project as a whole and that could not be attributed to a specific activity, for instance electricity, some of the office supplies, communication, fuel, car maintenance costs. | Same as in TaNT, except for consumables that could eventually be purchased for a lower price by the MoH (such as field material). |

## **Appendix 2. Input categories**

Tables S2 and S4 present the lists of input items used in the data collection and cost calculation, adapted from the DOLF (Death to Onchocerciasis and Lymphatic Filariasis) project: Protocol for Cost Data Collection in Community Trials.[9] Tables S3 and S5 present the costs of the input items responsible for the highest shares of the total costs, per input category and activity.

### **Supplementary Table S2. Inputs of supplies categories**

| **Supplies categories** | **Comment** |
| --- | --- |
| Electricity | Electricity bill of the building where the implementing institution is based, during the months of field work. |
| Office Supplies | Supplies used during the entire round. |
| Food Supplies | Coffee breaks during training. |
| Communication | Development of Information, Education and Communication (IEC) materials, media campaign and coverage, launching ceremony, IEC materials. |
| Fuel | Fuel used by the cars transporting personnel and/or material. |
| Car maintenance | Maintenance of the cars used in the round. |
| LoaScope kits (15) | The costs of each LoaScope (including cell phone, lens, charger) was US$ 700, and 15 LoaScopes were used in this project. The estimated average useful lifetime of a LoaScope was assumed to be 5 years, as suggested by the manufacturer. Since we are calculating the costs of the first year of a TaNT strategy using LoaScopes, the costs were divided by 5 and not discounted or annuitized. |
| Adverse reaction drugs | Drugs purchased by the implementing institution especially for the treatment of possible AEs during the round (more details in table S8). |
| Medical care/Hospitalization | When AEs eventually needed more than only drugs to be managed. |
| Field material | All material used in the field (more details in table S8). |
| Capillaries | Costs of the capillaries used were US$ 1.10 (unit price), and it was assumed that one capillary was used for each person tested. |
| Teaching material | Material used during training (paper, pens, chalk). |
| Other direct costs | Other direct costs include the following costs that could not be attributed to specific inputs: cleaning materials and toilet paper for field team, binding of registries, towel, cleaning of training room, per diem for driver to bring field waste to incineration site. |
| Sound car | Includes the preparation of 4x4 pick up car with megaphone, the fuel used only by the sound car and its maintenance. |

### **Supplementary Table S3. Costs of supplies responsible for the highest shares of the total costs disaggregated by input category and activity (US$)**

| **Supply Category** | **Quantity** | **Unit price (US$)** | **Total** | **1 Advocacy** | **2 Census** | **3 Planning and budgeting** | **4 Procurement** | **5 Training** | **6 Health education community / mobilization** | **7 Delivery intervention** | **8 Adverse event surveillance and management** | **9 Monitoring / Evaluation** | **10 General Management** | **% of  total pilot costs** |
| --- | --- | --- | --- | --- | --- | --- | --- | --- | --- | --- | --- | --- | --- | --- |
| Capillaries | 30,808 | 1.1 | 33,889 | - | - | - | ^a^ | - | - | 33,889 | - | - | - | 12% |
| IEC materials | ^b^ | ^b^ | 27,398 | 5,844 | 44 | 18 | ^a^ | 9 | 20,060 | 49 | 9 | - | 1,365 | 10% |
| Field material | ^b^ | ^b^ | 10,286 | - | 55 | - | ^a^ | - | - | 7,732 | - | - | 2,499 | 4% |
| Car maintenance (car/month) ^c^ | 47 | 146 | 8,160 | - | 24 | 2 | ^a^ | 10 | 162 | 22 | 7 | - | 7,934 | 3% |
| Fuel (liters) ^c^ | 7,614 | 129.6 | 7,260 | 176 | 618 | 53 | ^a^ | 863 | 106 | 2,691 | 1,220 | - | 1,533 | 3% |
| LoaScopes purchase | 15 | 700 | 2,100 ^d^ | - | - | - | ^a^ | - | - | 2,100 | - | - | - | 1% |
| Other categories | N.A. | N.A. | 8,735 |  |  | 27 | 30 | 1,655 | 1,114 | 44 | 812 | 107 | 4,948 | 3% |
| Total Supplies | N.A. | N.A. | 97,830 | 6,020 | 741 | 100 | 30 | 2,537 | 21,442 | 46,527 | 2,048 | 107 | 18,280 | 34% |

^a^ Procurement by administrative personnel (included under overhead costs)

^b^ Appendix 5 includes a table with the quantities of the main elements of IEC and field material.

^c^ Car maintenance and fuel costs given in average cost /car / month (7 vehicles during 8 months).

^d^ Total of LoaScopes purchase divided by 5 (assuming 5 years of useful lifetime).

IEC – Information, Education and Communication

N.A. – Not applicable

### **Supplementary Table S4. Inputs of personnel categories**

| **Inputs - Personnel categories** | **Comment** |
| --- | --- |
| Implementing institution | Salaries, fringe costs and/or per diems of manager, supervisors of training and field activities, general administration staff, team for information/sensitization at district level, and data management consultant, when applicable. |
| Ministry of Health (MoH) external staff^a^ | Salaries and/or per diems of national, district and health facility level staff. Per diems of community leaders/mobilizers. |
| Drivers | Per diems. |
| Sound Car | Per diems of the sensitization team (one driver, one former National Onchocerciasis Control Program coordinator (team lead) and one team member from CRFilMT). |
| School staff | Per diems of school staff helping during testing and treating of children in schools. |
| Administrative authorities | Per diems of administrative authorities during advocacy phase. |
| CDDs | Per diems. |
| Loascopists and blood drawers | Per diems. |

^a^ The salaries of the Minister of Health and the director of MoH’s Disease Control Division were not included, but apart from advocacy activities, they are not usually in the budget, so the impact on the total cost is assumed to be very small.

### **Supplementary Table S5. Costs of personnel disaggregated by category and activity (US$)**

| **Personnel Category** | **#** | **Per diem days** | **Per diem (US$/day)** | **Total per diems** | **Salaries and  fringe costs** | **Total payments** | **1 Advocacy** | **2 Census** | **3 Planning and budgeting** | **4 Procurement** | **5 Training** | **6 Health education community / mobilisation** | **7 Delivery intervention** | **8  Adverse event surveillance & management** | **9 Monitoring / Evaluation** | **10 General Management** | **% of total pilot costs** |
| --- | --- | --- | --- | --- | --- | --- | --- | --- | --- | --- | --- | --- | --- | --- | --- | --- | --- |
| **Implementing institution** | | | | | | | | | | | | | | | | | |
| Manager | 1 | 53 | 70 | 3,699 | - | 3,699 | - | - | - | * | - | - | - | - | - | 3,699 | 1% |
| Supervision of field activities | 14 | 983 | 70 | 69,277 | 6,336 | 75,614 | - | 9,948 |  | * | 6,644 | - | 20,183 | 9,026 | - | 29,813 | 26% |
| General Administration | 2 | 63 | 70 | 4,438 | - | 4,438 | - | - | 4,438 | * | - | - | - | - | - | - | 2% |
| IEC team | 3 | 26 | 44 | 1,154 | - | 1,154 | - | - | 1,154 | * | - | - | - | - | - | - | 0·4% |
| Data management consultant | 1 | 570 | 18 | 10,039 | - | 10,039 | - | - | - | * | - | - | - | - | 10,039 | - | 4% |
| **MoH** | | | | | | | | | | | | | | | | | |
| MoH national level | 5 | 114 | 53 | 6,030 | 623 | 6,653 | 402 | 1,177 | 1,559 | 9 | 1,514 | - | 1,975 | - | 18 | - | 2% |
| MoH district and health area levels | 8 | 44 | 26 | 2,575 | 1,413 | 3,988 | 63 | 385 | 26 | * | 132 | 173 | 486 | 117 | 92 | 1,101 | 1% |
| **Other** | | | | | | | | | | | | | | | | | |
| CDDs | 600 | 4,153 | 4 | 18,287 |  | 18,287 | 35 | 6,006 |  | * | 3,538 |  | 7,343 |  | 1,365 |  | 6% |
| Drivers | 7 | 349 | 26 | 9,211 | - | 9,211 | - | 1,459 | - | * | 796 | 1,198 | 4,649 | 1,110 | - | - | 3% |
| Loascopists & blood drawers | 100 | 1,434 | 5 | 7,579 | - | 7,579 | 26 | - | 48 | * | 918 | - | 6,494 | - | 92 | - | 3% |
| Various personnel | N.A. | N.A. | N.A. | 9,779 |  | 9,779 | 317 | - | 845 | * | - | 7,608 | 1,008 | - | - | - | 3% |
| **Total** | N.A. | N.A. | N.A. | 140,655 | 8,372 | 149,027 | 843 | 18,975 | 8,071 | 9 | 13,542 | 8,979 | 42,137 | 10,253 | 11,606 | 34,613 | 52% |

# Number of professionals

* Procurement by administrative personnel (included under overhead costs)

IEC team - Information, Education and Communication

MoH – Ministry of Health

CDDs – Community Drug Distributors

N.A. - Not applicable

Personnel were paid per diems for days of work outside their working place (receiving/giving training or any days of field work). To the costs of the per diems were added the costs of the daily salaries of each professional, to account for the opportunity cost of their time not spent in other projects. The structure needed for the use of the LoaScope still does not allow a house-to-house strategy, so we kept the costs of using mobile stations.

Overhead costs were included to capture the shares of the salaries of the administrative and financial managers (general administration), and the coordinator, building rental, cleaning and security services attributable to this project. Capital items are here defined as having a life expectancy of more than 5 years. Capital items such as vehicles, computers, software, office furniture, communication or audiovisual equipment used in the Soa campaign had already been paid by previous projects (except for the LoaScopes, no capital items were purchased specifically for this project) and were not included in the cost calculations. The variable running costs related to such capital items were explicitly included in the micro-costing study. Overhead costs were billed to the funding source (Bill and Melinda Gates Foundation via Institut Bouisson-Bertrand) as 15% of all actual expenditures of the entire round (of all costs of supplies and personnel). This seems to be a reasonable estimate of the overhead costs, considering that the TaNT campaign is not the only project to fund these expenses. These resources are jointly used by more programmes run by CRFilMT. A higher percentage bears the risk of double counting.

Costs exclusively related to research (research protocol driven costs) were not included in this study. They include costs related to travelling to and attending research meetings, congresses, research visits from international researchers for the development of data collection instruments, costs of all researchers working on the cost collection and calculations.

## **Appendix 3. Questionnaires**

Questionnaires were adapted from those used in the DOLF project.[9]

A prospective questionnaire with a user-friendly design with tables, tick boxes and expected short answers was chosen based on feedback from the CDDs. This was used to investigate the number of hours CDDs spent on transportation, training, census-taking, treatment and reporting. It was also used to collect information about their occupation and income, capture their opportunity costs and enable the use of real information instead of arbitrary valuation systems such as Gross National Income (GNI) or rural wage as a proxy for an 8-hour day of volunteer labor.[10]

The English versions of the French questionnaires are included below.

### **Supplementary Table S6. Design, structure and dissemination of the questionnaires for CDDs and external staff from the Ministry of Health.**

| **Questionnaire number and target** | **Content** | **Type** | **Number of pilot tests, pilot test dates and area** | **Dissemination method and time** | **Completion period** |
| --- | --- | --- | --- | --- | --- |
| 1. CDDs | Commuting time and costs to training, time spent in training, professional activity and income | Prospective | 3 rounds of about 20 CDDs, October 2017, in Ting Melen, Koulou and Ngali health areas | Printed and distributed at the end of the training session | At the end of each CDD training session |
| 2. CDDs | Education level, time spent for sensitisation, census and treatment, satisfaction regarding financial incentives | Prospective | 3 rounds of about 20 CDDs, October 2017, in Ting Melen, Koulou and Ngali health areas | Printed and distributed at the end of the training session | At the end of each CDD treatment campaign |
| 3. External staff from Ministry of Health (different levels) | Time spent on the TaNT project in Soa with break down by activity type, monthly salary, number and amount of received per diem | Retrospective | No pilot tests were carried out. | Applied individually during personal interviews | At the end of the TaNT round |

**Questionnaire 1**

**
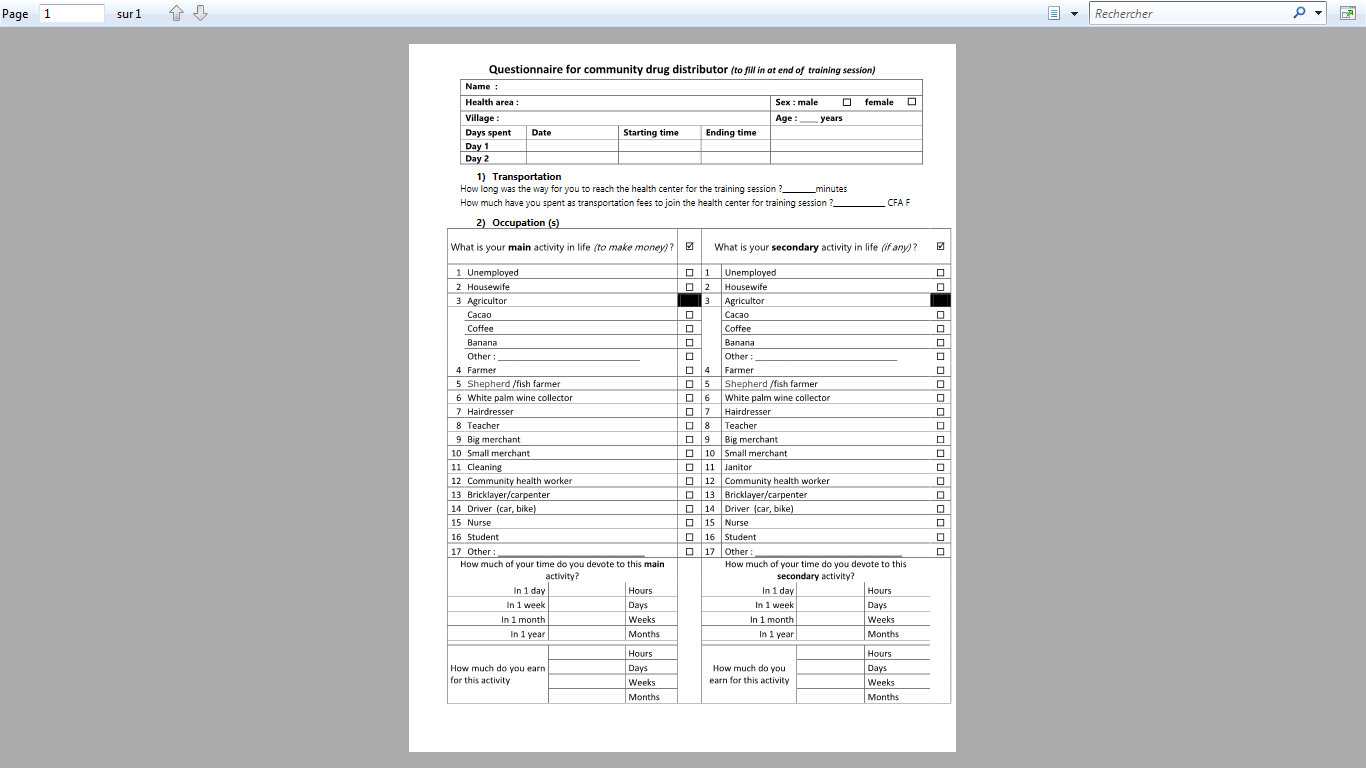
**

**Questionnaire 2**
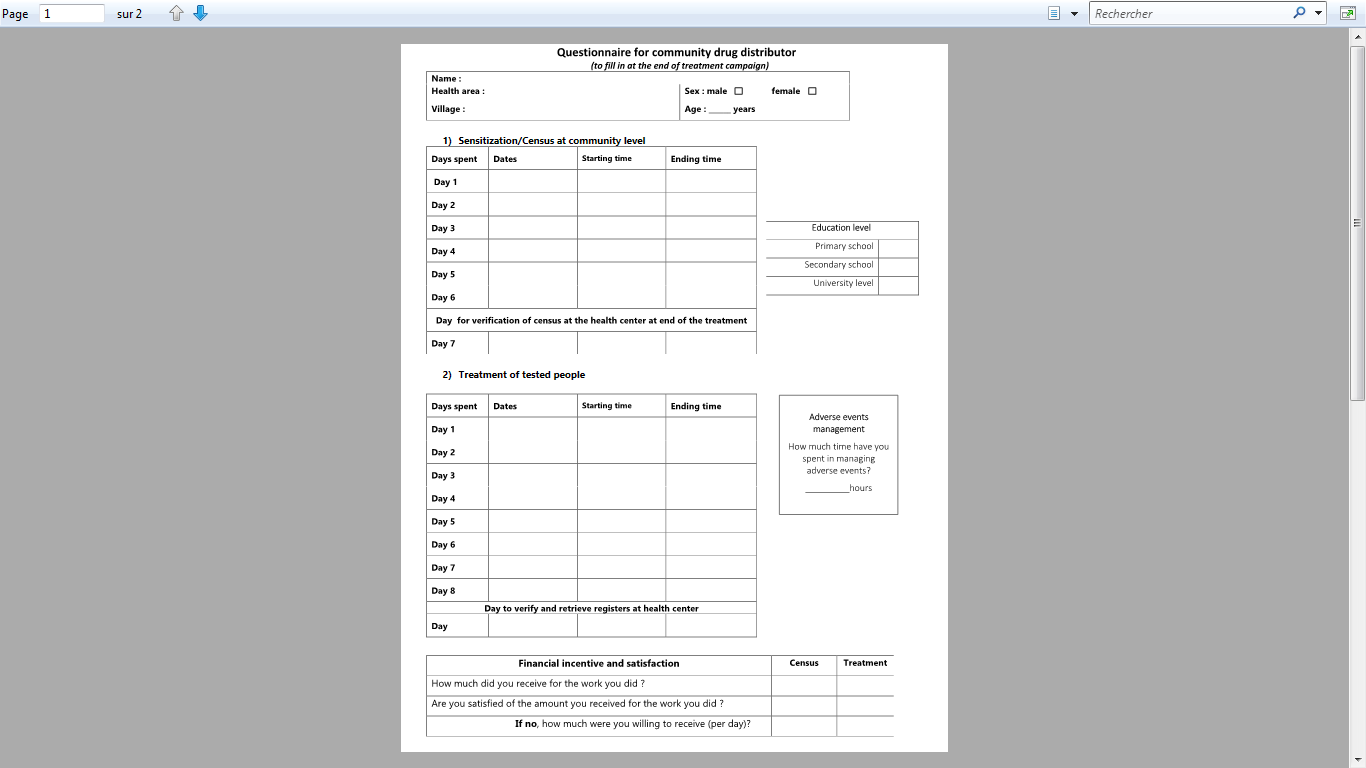


**Questionnaire 3**

## **Appendix 4. Scenarios**

The scenarios were designed according to the literature and our own expertise in conducting CDTI rounds in Cameroon, using the same health district as context.[7,8]

### **Supplementary Table S7. Assumptions regarding differences in personnel costs between the base case, less intensive and more intensive resource use implementation scenarios. Costs are specified in *Communauté Financière Africaine* (CFA, African Financial Community) francs (XAF).**

| **Input** | **Base case scenario** | | **Less intensive resource use scenario** | | | **More intensive resource use scenario** | | |
| --- | --- | --- | --- | --- | --- | --- | --- | --- |
| **Supervision of census, training and treatment^a^** | | | | | | | | |
| **Personnel type** | - - **Number of persons** | - - **Number of days per person** | **Number of persons** | **Number of days per person** | | - - **Number of persons** | - - **Number of days per person** | |
| National level | - - 1 | - - 6 days:   - 1 – receiving training,   - 2 – giving training,   - 1 – census,   - 2 – treatment | Same as base case. | | | **2** | Same as base case. | |
| Regional level | - - 1 | - - 7 days:   - 2 – receiving training,   - 2 – giving training,   - 1 – census,   - 2 – treatment | Same as base case. | | | **2** | Same as base case. | |
| District level | - - 2 | - - 7 days:   - 2 – receiving training,   - 2 – giving training,   - 1 – census,   - 2 – treatment | Same as base case. | | | **3** | Same as base case. | |
| Health area level | - - 6 chiefs of centers per District (on average) | - - 11 days:   - 2 – receiving training,   - 1 – giving training,   - 3 – census,   - 5 – treatment | Same as base case. | | | Same as base case. | Same as base case. | |
| NGDO (manager) | - - 1 | - - 5 days:   - 2 – giving training,   - 1 – census,   - 2 – treatment | Same as base case. | | | Same as base case. | Same as base case. | |
| - - NGDO   (supervisors) | - - 2 | - - 6 days:   - 1 – receiving training,   - 2 – giving training,   - 1 – census,   - 2 – treatment | Same as base case. | | | Same as base case. | Same as base case. | |
| - - Drivers | - - 5^b^ | - - 24 days:^c^   - 4 – receiving training   - 8 – giving training   - 4 – census   - 8 – treatment | Same as base case. | | | 7^b^ | Same as base case. | |
| - - CDDs | - - 716 (1/100 people) | 2 days  2 – receiving training  Census and treatment days not included in the calculations (not paid). | Same as base case. | | | Same as base case. | 5 days  2 – receiving training  3 – treatment  Census days not included in the calculations (not paid). | |
| - - Loascopists and blood drawers. | - - 478 (1/300 people) | 5 days  2 – receiving training  3 – treatment | Same as base case. | | | Same as base case. | Same as base case. | |
| Monitoring & evaluation (M&E) | | | | | | | | |
| National level | 1 | 4 days supervision of data collection | Same as base case. | | 3 days supervision of data collection | Same as base case. | | 6 days supervision of data collection |
| Regional level | 2 | 4 days compiling it in Excel + 4 days supervision of data collection | Same as base case. | | 3 days compiling it in Excel + 3 days supervision of data collection | Same as base case. | | 6 days compiling it in Excel + 6 days supervision of data collection |
| District level | 2 | 4 days compiling it in Excel + 4 days supervision of data collection | Same as base case. | | 3 days compiling it in Excel + 3 days supervision of data collection | Same as base case. | | 6 days compiling it in Excel + 6 days supervision of data collection |
| Chief of each health area | 6 | 4 days synthesizing data from the registers brought by CDDs + 4 days supervision of data collection | Same as base case. | | 3 days synthesizing data from the registers brought by CDDs + 3 days supervision of data collection | Same as base case. | | 6 days synthesizing data from the registers brought by CDDs + 6 days supervision of data collection |
| NGDO | 2 | 4 days supervision of data collection + data manager | Same as base case. | | 3 days supervision of data collection + data manager | Same as base case. | | 6 days supervision of data collection + data manager |
| Drivers (NGDO, Central level and Regional level) | 5^b^ | 20 days ^c^ | Same as base case. | | 15 days ^c^ | 7^b^ | | 42 days ^c^ |
| Advocacy | Same as pilot. | | Same as pilot. | | | Same as pilot. | | |
| Planning and budgeting | Same as pilot. | | Same as pilot. | | | Same as pilot. | | |
| Procurement | Same as pilot. | | Same as pilot. | | | Same as pilot. | | |
| Health education / community mobilization | Same as pilot excluding all costs related to the sound car (same as CDTI). | | Same as base case. | | | Same as base case. | | |
| Adverse event surveillance and management | Same as pilot. | | Assumed half of pilot costs. | | | Same as pilot. | | |

^a^ Extra supervision costs by the implementing institution during the pilot were excluded from all scenarios.

^b^ Total number of drivers for MoH personnel.

^c^ Total number of days transporting MoH personnel.

### **Supplementary Table S8. Assumptions regarding differences in personnel per diems between the base case, less intensive and more intensive resource use implementation scenarios. Costs are specified in *Communauté Financière Africaine* (CFA, African Financial Community) francs (XAF).**

| **Personnel type** | **Base case scenario** | **Less intensive resource use scenario** | **More intensive resource use scenario** |
| --- | --- | --- | --- |
| National level | •5,000 transportation fee to receive training/day;  •40,000 per diem to give training;  •40,000 per diem / field day. | Same as base case. | Same amount paid per person as in base case, but for 2 professionals instead of 1. |
| Regional level | •5,000 transportation fee to receive training/day;  •25,000 per diem to give training;  •25,000 per diem / field day. | Same as base case. | Same amount paid per person as base case, but for 2 professionals instead of 1. |
| District level | •20,000 per diem to receive training + 10,000 transportation fee to receive training;  •15,000 per diem + 5,000 transportation fee/day to give training;  •15,000 per diem + 5,000 transportation fee / field day. | Same as base case. | Same amount paid per person as base case, but for 3 professionals instead of 2. |
| Health area level | •10,000 per diem to receive training + 3,000 transportation fee/day to receive training;  •5,000 per diem + 3,000 transportation fee/day to give training;  •10,000 per diem + 2,000 transportation fee / field day. | Same as base case. | Same as base case.  3,000 transportation fee to give/receive training/day;  3,000 transportation fee / field day. |
| NGDO manager | • 40,000 per diem to give training;  • 40,000 per diem / field supervision day. | Same as base case. | Same as base case. |
| NGDO supervision | • 40,000 per diem to give training;  • 40,000 per diem / field supervision day. | Same as base case. | Same as base case. |
| CDDs (1 CDD / 100 inhabitants) | • 2,000 per diem + 500 transportation fee/day for 2 training days;  • not paid any per diems for census or treatment days. | Same as base case. | •3,000 per diem + 500 transport fee for each of 2 training days;  • 5,700 per diem / field day (average income for an eight-hour day (to account for income loss), according the CDDs’ responses in the questionnaires). |
| Blood drawers and Loascopists (1 of each / 300 inhabitants) | • 2,500 per diem + 600 transportation fee for each of 2 training days;  • 3,000 per diem + 600 transportation fee for each of the 3 treatment days | Same as base case. | •2,500 per diem + 4,200 transport fee for each of 2 training days;  •3,000 per diem + 4,200 transport fee for each of the 3 treatment days.  (transportation fee = two-way maximum transportation costs to training sites, based on CDDs’ responses) |
| Drivers (for national and regional levels and NGDO) | • 20,000 per diem | Same as base case. | Same per diem paid as base case, but more personnel (as shown above). |
| School staff | School staff not paid. | Same as base case. | 1 school staff per community, assume 100 community in need of school treatment per district;  5,000 per diem for 1 day per staff. |

### **Supplementary Table S9. Assumptions regarding differences in supplies costs between the base case, less intensive and more intensive resource use implementation scenarios. Costs are specified in *Communauté Financière Africaine* (CFA, African Financial Community) francs (XAF).**

| **Input** | **Base case scenario** | **Less intensive resource use scenario** | **More intensive resource use scenario** |
| --- | --- | --- | --- |
| Fuel | •30,000 XAF fuel allowance/day for central level and NGDO cars; 20,000 XAF fuel allowance /day for cars driven by regional level personnel; 10,000 XAF fuel allowance for district staff per field day | Same as base case. | 40,000 XAF fuel allowance per day for Central level cars, paid to more personnel (as shown above). |
| Consumables likely to have different prices when purchased by MoH (office supplies, communication tools, car maintenance, adverse reaction drugs, field material, teaching material, other direct costs). | Assumed 30% less compared to the costs of consumables of actual TaNT pilot. | Assumed 50% less compared to the costs of consumables of actual TaNT pilot. | Same as TaNT pilot. |
| LoaScopes purchase price | US$ 400 unit price (large-scale manufacturer’s prediction price). | Same as base case. | US$ 700 each (same as actual TaNT pilot). |
| Capillaries used for testing | US$ 0.40 unit price (large-scale manufacturer’s prediction price). | Same as base case. | US$ 1.10 each (same as actual TaNT pilot). |

**Fuel and drivers**. CRFilMT transported blood drawers and loascopists from their villages to the villages they were going to work at. In a CDTI round organized by the health district this would not be possible and it also decreases the efficiency of the work, since it delays the starting time in the field. To account for this, we added a transportation fee of 600 XAF to the per diems paid to blood drawers and loascopists in the base case scenario, using the average transportation cost paid by CDDs in our project to get to the trainings. They would still have to bring back LoaScopes to health centers at the end of each treatment day in order to recharge their batteries and check for any malfunction. Since personnel from the different levels would still need drivers to take them to training sites and to the field for supervision, we assigned one driver per person of the above-mentioned supervision team, for each day of work, with a per diem of 20,000 XAF. Since it is more difficult to have available drivers for district level staff, a fuel fee of 10,000 XAF was assigned to each of their days of work. Fuel allowance was also added to national and regional level personnel: 30,000 and 20,000 XAF, respectively. The sum of the per diem costs of drivers just mentioned replaced the costs of drivers’ per diems of the original TaNT pilot for census, training and delivery of intervention. Fuel costs were kept as the original round costs, added the costs of the fuel for the national, regional and district personnel.

Since we are considering a first implementation round in the scenarios, health education and community mobilization costs would be similarly high. Unlike during the pilot, a sound car is not typical. Thus, we excluded all costs relative to its use in all scenarios.

Personnel costs related to administrative authorities (180,000 XAF) were kept the same.

## **Appendix 5. Volumes of supplies input categories**

### **Supplementary Table S10. Volumes of supplies per input category. Prices specified in *Communauté Financière Africaine* (CFA, African Financial Community) francs (XAF).**

| **Item** | **Quantity** | **Unit price** | **Total price** |
| --- | --- | --- | --- |
| **Personnel material (variable)** | | | |
| Capillaries (receipt in Dec 2017) | 66,274 | 1.1 | 72,901 |
| LoaScopes purchase | 15 | 212,000 | 3,180,000 |
| Badges | 566 | 263 | 149,000 |
| Bags for blood drawers | 15 | 5,000 | 75,000 |
| T-shirts | 700 | 1,800 | 1,260,000 |
| **Drugs for AE management (variable)** | | | |
| Aerius 5mg coated tablet/7 | 1 | 2,600 | 2,600 |
| Amoxicilline 500mg gel B/1000 | 1 | 29,000 | 29,000 |
| Artemether | 9 | 650 | 5,850 |
| Arthemether+lumefantrin 20/120mg tablet disp B/6 | 10 | 1,121 | 11,210 |
| Arthemether+lumefantrin 80mg/480mg B/6 tablet | 10 | 910 | 9,100 |
| Arthemether+lumefantrine 20/120mg tablet B/24 | 9 | 855 | 7,695 |
| B complex vitamin tablet B/1000 | 1 | 13,146 | 13,146 |
| Yellow Betadine dermatologic solution flacon /125ML | 1 | 1,550 | 1,550 |
| Chlorpheniramine B/10 | 60 | 500 | 30,000 |
| Cloxacilin | 6 | 500 | 3,000 |
| Compress 40*40 | 1 | 1,450 | 1,450 |
| Dexametazone | 6 | 100 | 600 |
| Diclofenac DENK 50 TABLET B/2x10 | 50 | 794 | 39,700 |
| DIFENASOL flacon /5ML | 10 | 680 | 6,800 |
| Dynapar 100mg | 1 | 950 | 950 |
| Pregnancy test | 1 | 500 | 500 |
| Gentamycin eyedrops 10mL | 5 | 525 | 2,625 |
| GENTASOL CY flacon 5mL B/1 | 10 | 713 | 7,130 |
| Glove | 3 | 100 | 300 |
| Glycaemia | 1 | 1,000 | 1,000 |
| Health care visit ticket | 1 | 300 | 300 |
| Ibuprofen 400 tablet B/10x10 | 5 | 1,050 | 5,250 |
| Ibuprofen 400mg B/100 tablet | 5 | 1,350 | 6,750 |
| Iron sulfate 200mg+Folic acid 0.25mg tablet B/1000 | 2 | 22,000 | 44,000 |
| Laritem 80mg/480mg tablet/6 | 1 | 2,650 | 2,650 |
| Loratadine 10mg B/10 tablet | 20 | 1,407 | 28,140 |
| Metronizadole 250mg tablet B/1000 (Flagyl) | 3 | 6,890 | 20,670 |
| Crepe band 4*7C | 1 | 600 | 600 |
| Paracetamol 500mg tablet B/100 | 100 | 425 | 42,500 |
| Patient card | 1 | 300 | 300 |
| Quinine sulfate 300mg tablet B/1000 | 1 | 32,400 | 32,400 |
| Plaster 5x5cm | 1 | 2,550 | 2,550 |
| Syringe | 6 | 100 | 600 |
| Vitamin B complex tablet B/1000 | 2 | 13,146 | 26,292 |
| Voltaren emulgel 1% T 50G | 1 | 1,875 | 1,875 |
| Hemoglobin test | 1 | 500 | 500 |
|  |  |  |  |
| **Fuel and car maintenance (variable)** | | | |
| Car maintenance (episodes) | 47 | 98,584 | 4,633,429 |
| Fuel (Liters) | 7,144 | 577 | 4,121,900 |
| **IEC materials and registers (variable)** | | | |
| IEC Leaflets | 10,000 | 170 | 1,700,000 |
| IEC Posters | 1,000 | 2,500 | 2,500,000 |
| Registers | 900 | 2,777 | 2,499,000 |
| **Field materials (variable)** | | | |
| Alcohol 95% (liters) | 200 | 1,500 | 300,000 |
| Bin bag 50Lx20 | 11 | 1,425 | 15,675 |
| Bleach water (1 liter) | 1 | 950 | 950 |
| Bleach water 250ml | 10 | 300 | 3,000 |
| Hydrophilic cotton (pack of 500g) | 130 | 1,900 | 247,000 |
| Disposable cup | 127 | 1,400 | 177,800 |
| Chalk (pack of 100) | 23 | 1,614 | 37,129 |
| Gloves (pack of 20) | 86 | 25,000 | 2,150,000 |
| Grapefruit anti-bac | 10 | 1,000 | 10,000 |
| Hand washing gel 500ml | 9 | 975 | 8,775 |
| Lancets (pack of 200) | 120 | 3,000 | 360,000 |
| Paper towels | 21 | 3,000 | 63,000 |
| Paper towels large size*2 | 5 | 1,500 | 7,500 |
| Paper towels large size*6 | 5 | 2,400 | 12,000 |
| Mineral water (10L) | 5 | 1,250 | 6,250 |
| Recycle bin bags | 20 | 1,750 | 35,000 |
| Trash bucket | 1 | 1,500 | 1,500 |
| Tablecloth (pack) | 1 | 14,000 | 14,000 |
| Wraps (pack of 72) | 21 | 2,350 | 49,350 |
| **Office supplies (fixed)** | | | |
| Staple remover | 1 | 500 | 500 |
| Paper (reams of 500 sheets) | 117 | 2,250 | 263,250 |
| Pen (pack of 144) | 4 | 40,750 | 163,000 |
| Pencil | 60 | 50 | 3,000 |
| Pencil sharpener | 20 | 150 | 3,000 |
| File with 40 plastic bags | 1 | 1,600 | 1,600 |
| File with 40 plastic bags | 1 | 1,850 | 1,850 |
| Printer cartridge | 14 | 47,000 | 653,000 |
| Paper clips (1000) | 1 | 2,000 | 2,000 |
| Air time (global) | 70 | 5,000 | 348,000 |

##

## **Appendix 6. Benchmark tool use**

Fitzpatrick et al. (2016) developed a web-based software application that allows each user to calculate MDA round costs per person treated, varying different aspects according to each context.[12] We used the tool for the calculation of economic costs with a population of 71,643 (our censused total population), coverage rate of 42% (our final coverage rate), subnational, with no school-based delivery, no volunteers, 1 disease (stand-alone program), 1 round per year, first year of implementation, GDP per capita (2017) of US$ 1446.70 [13], and population density of 51.91/sq km [14] for the setting. We chose McFarland and Menzies (2005) for study-specific fixed effect, whose methodology was comparable to ours.[10]

## **References**

1. Zoure HG, Wanji S, Noma M, Amazigo UV, Diggle PJ, Tekle AH, et al. The geographic distribution of Loa loa in Africa: results of large-scale implementation of the Rapid Assessment Procedure for Loiasis (RAPLOA). PLoS Negl Trop Dis 2011;5(6):e1210.

2. World Health Organization. Expanded Special Project For Elimination of Neglected Tropical Diseases -  Cameroon. 2018; Available at: <http://espen.afro.who.int/countries/cameroon>. Accessed Aug/10, 2018.

3. Sodahlon YK. SAEs Following Onchocerciasis Treatment with ivermectin - Mectizan Donation Program. 51th MEC/AC Meeting - Seattle 2014.

4. D'Ambrosio MV, Bakalar M, Bennuru S, Reber C, Skandarajah A, Nilsson L, et al. Point-of-care quantification of blood-borne filarial parasites with a mobile phone microscope. Sci Transl Med 2015;7(286):286re4.

5. Kamgno J, Pion SD, Chesnais CB, Bakalar MH, D'Ambrosio MV, Mackenzie CD, et al. A Test-and-Not-Treat Strategy for Onchocerciasis in Loa loa-Endemic Areas. N Engl J Med 2017;377(21):2044-2052.

6. Gardon J, Gardon-Wendel N, Demanga-Ngangue, Kamgno J, Chippaux JP, Boussinesq M. Serious reactions after mass treatment of onchocerciasis with ivermectin in an area endemic for Loa loa infection. Lancet 1997;350(9070):18-22.

7. Meredith SE, Cross C, Amazigo UV. Empowering communities in combating river blindness and the role of NGOs: case studies from Cameroon, Mali, Nigeria, and Uganda. Health Res Policy Syst 2012;10:16-4505-10-16.

8. UNDP / World Bank / WHO. Special Programme for Research and Training in Tropical Diseases (TDR). Community Directed Treatment with Ivermectin: report of a multi-country study. Applied Field Research Reports 1996;TDR/AFR/RP/96.1(<http://www.who.int/tdr/publications/documents/comdti_1.pdf.>).

9. Goldman AS. DOLF PROJECT: Protocol for Cost Data Collection in Community Trials - 2X vs 1X Annual MDA. Washington, DC, USA: The George Washington University School of Public Health and Health Services; 2010.

10. McFarland D, Menzies D, Njoumemi Z, et al. Study of cost per treatment with ivermectin using the CDTI strategy. African Programme for Onchocerciasis Control (APOC) 2005.

11. Bureau of the Fiscal Service, US Department of the Treasury. Treasury Reporting Rates of Exchange. 2018; Available at: <https://www.fiscal.treasury.gov/fsreports/rpt/treasRptRateExch/historicalRates.htm>. Accessed March/20th, 2018.

12. Fitzpatrick C, Fleming FM, Madin-Warburton M, Schneider T, Meheus F, Asiedu K, et al. Benchmarking the Cost per Person of Mass Treatment for Selected Neglected Tropical Diseases: An Approach Based on Literature Review and Meta-regression with Web-Based Software Application. PLoS Negl Trop Dis 2016;10(12):e0005037.

13. World Bank. GDP per capita (current US$). 2018; Available at: <https://data.worldbank.org/indicator/NY.GDP.PCAP.CD>. Accessed September/22, 2018.

14. World Population Review. Cameroon population. 2018; Available at: <http://worldpopulationreview.com/countries/cameroon-population/>. Accessed September/22, 2018.
